# Some Practical Points in the Physiology and Pathology of the Fifth Pair of Nerves in Relation to Inveterate Neuralgia

**Published:** 1888-06

**Authors:** Stretch Dowse


ARTICLE III.

### SOME PRACTICAL POINTS IN THE PHYSIOLOGY AND PATHOLOGY OF THE FIFTH PAIR OF NERVES IN RELATION TO INVETERATE NEURALGIA.

BY DR. STRETCH DOWSE.

He did not propose either to follow in the footsteps or tread on the heels of Mr. Victor Horsley's paper of the previous session, which dealt almost exclusively with the surgical aspect of the question, but in the belief that all cases were not adapted to surgical interference, rather to invite attention to and provoke discussion on the medical phase of the subject.

Having somewhat fully reviewed the latest knowledge obtained, both anatomically, physiologically and pathologically; and having quoted one or two cases bearing upon the question—notably a very important, and, as it were, test case of paralysis of the fifth cranial nerve, sensory and motor, published by Dr. Ferrier in the *Lancet* of January 7th, 1888, and bristling with clinical acumen and physiological knowledge—he entered upon what he regarded as the most interesting part of his paper, prefacing it with a reference to the trophic influence of the fifth nerve * They were all possibly aware of the existence of a disease known as loco-motor ataxy, but it was necessary to state that ataxy in locomotion did not always exist. There were many objective signs and symptoms, however, connected with the disease which should be of the greatest possible importance to the dentist. The fifth nerve, it must not be forgotten, was a spinal nerve, and its sensory connections were so extensive, and its reflex relations so vast, that it was hardly possible to conceive an essentially sensory disease like that of loco-motor ataxy existing unless the fifth nerve were involved. The involvement of the fifth nerve in loco-motor ataxy had never been recognized to the extent that he (Dr. Dowse) thought it ought to be. It did not seem to have struck neurologists that inveterate neuralgias of the fifth nerve were in part due to sclerosis of the ascending fibres which run into the trigeminus from the posthorn of the cervical spinal cord. He was visiting within an hour of each other two patients; one was suffering from the lightning pains of loco-motor ataxy and rubbing the skin from his thigh, so great was the agony; the other was suffering severely from tic of the gums and parts supplied by the infra-orbital nerve. The definition of the pain in each case was precisely similar; it came without the slightest warning, and was of the most intense character, and left as it came. There could be no doubt in his mind as to the absolute relationship between these two pains,—

---

* The trophic root of the trigeminal nerve arose from a mass of cells at the side of the aqueduct of Sylvius, or, as some would say, the trophic fibres originated in the gasserian ganglion itself. It was clear that they had much to do with the gums and their mucous membrane, and with the tooth pulp.

viz., of loco-motor ataxy and of facial tic, with or without muscular spasm, known as tic-douloureux.

The most prominent signs of loco-motor ataxy were—ataxic movements; fulgurating pains; localised hyperæsthesia; ocular paralysis; numbness and other dysæsthesia; anæsthesia; staggering with closed eyes; failure of sexual power; absence of tendon reflex; rectal and vesical paueses; gastric crises; laryngeal crises; vesical crises; severe arthropathies; amaurosis; complicating transverse myelitis; spinal congestion; paralytic dementia; vesical catarrh; pupillary changes.

Of these signs he would only draw their attention to (1), pupillary changes; (2), ataxic movements; (3,) fulgurating pains; (4), severe arthropies; (5), absence of tendon reflexes.

(1.) In ataxy the pupils were invariably very small, dilated sluggishly if at all, but contracted when the eye was accommodated for a near object; again, one pupil might be very large, while the other might be very much contracted.

(2.) The patient was unable to maintain the equilibrium in the dark, or when he was made to stand erect with the eyes closed, or he felt himself losing his balance when washing his face.

(3.) Fulgurating pains were, in his experience, very diagnostic of the disease, and he had called attention to the fact that these fulgurating pains were in their character and intensity precisely analogous to the pains of the face due to the fifth nerve, and known as tic. He would ask, was inveterate neuralgia of the face due to sclerosis of the cervical branch of the fifth nerve, to its nuclei of origin in the medulla, to its connection with the sensory trigeminal nucleus which lies at the level of the pons, or to its ganglia? or, on the other hand, was it essentially peripheral?

He maintained, from analogy, that the lightning pains of the limbs, which were considered as common to sclerosis of the posterior columns of the spinal cord, were identical in character and causation to those pains of the sensory division of the fifth nerve, and known as tic of the face, epileptic neuralgia and tic-douloureux.

(4.) Associated with some class of loco-motor ataxy were found a peculiar state of the joints, known as Charcot's joints. The pain was characterised usually by enormous swelling from effusion into the joint. It was quite painless, even upon active movement, due to insensibility of the structures with wearing away of the articular ends of the bones. He would ask, was there anything analogous to this in the alveolar processes of the jaws, as well as the tooth fangs or dental pulp?

(5.) The pupillary changes might be accompanied with other phenomena, known as tendon reflexes, the most common of which, and the only one to which he cared to call attention, was absence of the "knee reflex." This was elicited by striking the patella tendon below the knee with the tips of the fingers when the leg is semi-flexed, or when one leg is crossed over the other. In health, when this tendon was tapped, the leg was thrown forwards and upwards, but when sclerosis of the sensory channels of the spinal cord existed, the leg was absolutely immovable. He would not weary them further with the signs of loco-motor ataxy; the practical outcome of importance seemed to be in regard to the wholesale extraction of teeth. When in doubt as to the value of wholesale extraction, examine the pupil of the eye, the absence or presence of the knee reflex, and the balancing power of the patient when standing erect with the eyes closed. If the signs were present indicating sclerosis of sensory nerve fibres, or if there were marked hysteria, unlimited extraction would not seem to be justifiable; if, on the other hand, these signs were absent showing that peripheral irritation was the cause of pain, then, and then only, could any amount of operative interference be recommended as likely to be of benefit.

As to treatment of neuralgia of the fifth nerve, Dr. Dowse quite endorsed Professor Horsley's opinion with regard to "nerve-stretching," but as to "avulsion" he was somewhat sceptical. The best advice which he (Dr. Dowse) could give was to be found in the thoughtful and experienced remarks of their past learned President, Mr. Tomes, in the discussion of Mr. Horsley's paper, "Relief from severe and persistent pain quite justifies any ordinary surgical procedure, but I cannot quite see how relief means cure. How often do we see this word written to a case when the word relief would be far more appropriate."

In the treatment of neuralgia, as far as drugs were concerned, Dr. Dowse put great faith in the value of opium, when it agreed. Broadly, he treated facial neuralgias by drugs as follows :—When of the supra-orbital nerve, 20 or even 30 gr. doses quinine, just before the time for the paroxysm to make its appearance, and every other night a pill of calomel and gambooge, a plain non-alcoholic diet and plenty of milk. When of the middle and inferior maxillary nerves, he gave iodide and bromide of potassium, with 1-2 drachm doses of gelsemium three times a day. M. Goubler stated at the Societe Therapeutique, January 25th, 1877, that he did not know of a neuralgia of the fifth nerve, even tic-doloureux, which had resisted aconotine. Whilst speaking of drugs in neuralgia of the fifth nerve and in the fulgurant pains of ataxy, he (Dr. Dowse) had been much pleased with the effect of antipyrine in one scruple doses every half-hour until the pain was relieved.

He had for some time injected an ethereal solution of ergotine with marked good effects in trigeminial neuralgia and sciatica. He usually injected 1 gr. ergotine dissolved in 5 mins. ether; in facial neuralgia he injected into the scalp immediately behind the ear, and in sciatica he pushed the needle of the syringe well into the sciatica nerve. He had used a 1 per cent. solution osmic acid in the same way with much the same result. Dr. Dowse referred to a pamphlet by J. Montaign Didsbury, translated by Mr. Boyd-Wallis, on "The Treatment of Inveterate Neuralgia of the Fifth Nerve," advocating the division and cauterization with the actual cautery the auriculo-temporal nerve, on the assumption that "it is the shock to the grey axis of the medulla, produced by cauterization, that effects the cure; the application is made to the helix of the ear." Dr. Dowse thought there was much to be said in favour of the treatment.

Finally, a few words on the application of galvanism. He preferred the voltaic or galvanic current to Faradization —more frequently he combined the two, the voltaic current being in excess cf the Faradaic. He never used strong currents, 2, 3, or 4 milliampere of the continuous current being sufficient. For neuralgia of the first division of the fifth nerve, he plugged the nostril with very soft sponge or cotton wool, and into this he inserted a fine wire electrode (positive pole), which he fixed the negative electrode in the form of a long plate to the service-dorsal region of the spinal cord, and then completed the circuit. The current should be passed ten, twenty, or even thirty mins. at one sitting, and continued daily for a week after the pain had disappeared. Practice and discrimination was necessary in the application of the current. Faradization alone was worse than useless.—*London Dental Record.*

